# Supplementary material for: G protein-coupled estrogen receptor regulates embryonic heart rate in zebrafish
Source: PLoS Genet. 2017 Oct 24;13(10):e1007069. doi: 10.1371/journal.pgen.1007069 (PMC5669493; doi:10.1371/journal.pgen.1007069)

A

```
ATGGAGGAGCAGACTACCAATGTGATTCAGATTTATGTGAATGGCACCGAGCAGTTCAATGCTTCGTTTGACTTCAACATA
ACTGATGTGAAAGAAAGCACAGACACCTATGAGTTTTACATCATCGGCCTGTTTCTCTCATGCCTGTACACCATTTTCCTT
TTCCCAATTGGTTTCATTGGGAACATCCTCATTTTGGTGGTCAACCTCAACCACAGGGAGAGGATGACCATCCCAGATCTG
TACTTTGTCAACCTGGCTGTGGCAGATCTTATTCTGGTGGCAGACTCTCTCATTGAAGTCTTCAATCTCAACGAGAAGTAC
TACGACTATGCTGTACTGTGTACCTTCATGTCGCTTTTTCTCCAGGTAAACATGTATAGCAGCATCTTCTTCCTGACATGG
ATGAGTTTCGACCGCTACGTTGCTCTCACCAGCTCTATGAGCAGCAGTCCATTGCGAACCATGCAGCATGCCAAACTCAGT
TGCAGCCTCATCTGGATGGCCTCCATCCTGGCAACTCTGCTTCCTTTCACAATTGTGCAGACGCAACATACCGGTGAGGTG
CACTTCTGCTTCGCCAATGTCTTCGAGATCCAGTGGCTCGAGGTGACGATTGGATTTCTAATACCATTCTCCATTATCGGC
CTGTGCTACTCCCTGATTGTCCGCACTCTCATGCGTGCCCAGAAGCACAAGGGATTGTGGCCTCGACGACAAAAGGCCCTG
CGCATGATTGTGGTGGTTGTCTTGGTGTTCTTCATCTGCTGGCTTCCCGAGAATGTCTTCATTAGCATCCAGCTACTCCAA
GGCACAGCCGATCCATCGAAGCGTACTGACACAACACTATGGCATGACTACCCTTTAACCGGGCACATTGTCAACCTCGCT
GCATTCTCCAACAGCTGCCTAAACCCAATAATTTACAGCTTCCTTGGGGAGACCTTCAGGGATAAGCTGCGTCTCTTCATT
AAGAGGAAGGCAAGCTGGTCTGTGGTCTACCGCTTCTGTAATCACACTCTGGACCTGCAGATCCCTGTCAGGAGTGAGTCT
GAGGTGTAG
```

B

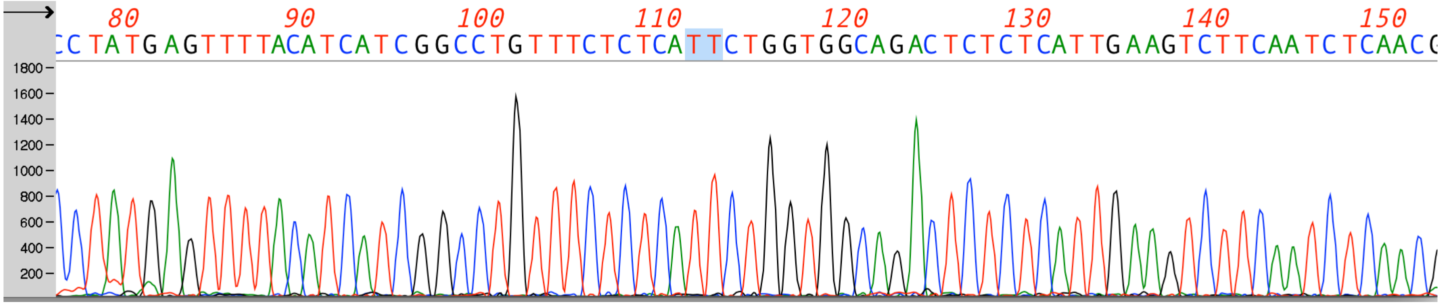

C

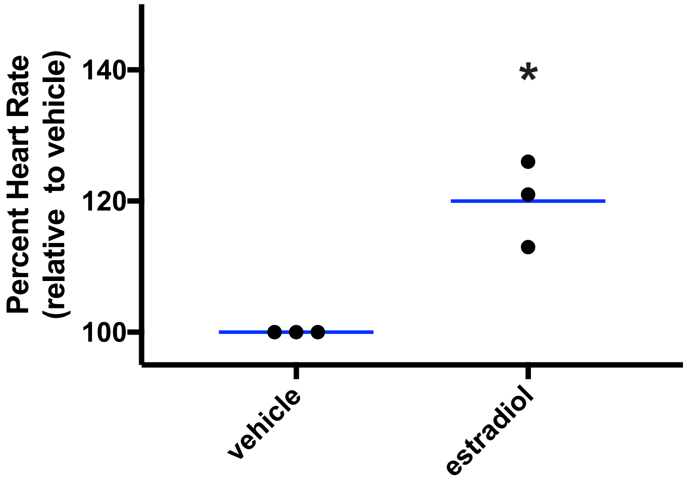

Supplement: S1 Fig — (A) Genomic DNA sequence of zebrafish gper open reading frame, contained in a single exon. Highlighted nucleotides are deleted in uab102. (B) Chromatogram of uab102 genomic DNA sequence. 133 basepair deletion occurred in between highlighted thymine (T) nucleotides. (C) Zygotic homozygous gper mutant embryos were incubated in water containing estradiol (ER/GPER agonist, 3.67 μM) or vehicle control (0.1% DMSO) at 49 hours post fertilization and heart rates were measured 1 hour post treatment. *, p<0.05 compared to vehicle, paired t test. Each black circle represents the mean heart rate from a single clutch of embryos (≥ 6 embryos per clutch). Clutches in the same treatment group were assayed on different days. Horizontal blue lines are the mean of each treatment. (PDF) [file pgen.1007069.s001.pdf]
