# Supplementary material for: G protein-coupled estrogen receptor regulates embryonic heart rate in zebrafish
Source: PLoS Genet. 2017 Oct 24;13(10):e1007069. doi: 10.1371/journal.pgen.1007069 (PMC5669493; doi:10.1371/journal.pgen.1007069)
Supplement: S2 Fig — (A) Genomic DNA of esr1uab118 zebrafish contains a 4 basepair deletion in the esr1 coding region, resulting in a premature stop codon in the Esr1 (ERα) protein. Nucleotide deletions are shown as red dashes, amino acid mutations are in red. Map indicates site of frameshift mutation and premature stop codon (AF-1, activating function 1 domain; DBD, DNA binding domain; LBD, ligand binding domain; AF-2, activating function 2 domain). (B) High resolution melting curve analysis was used to distinguish mutants from wildtype. Curves represents DNA amplified from a wildtype AB (black) or esr1uab118 mutant zebrafish (cyan). (C) Strategy for validating zebrafish estrogen receptor mutants using transgenic 5xERE:GFP zebrafish. Mutants were generated on a transgenic background where estrogen receptor (ER) transcriptional activity is marked by green fluorescent protein (GFP) expression. Following exposure to estradiol, loss-of-function mutants should exhibit reduced fluorescence in cells expressing esr1. (D-K) 2-day post fertilization embryos were exposed to 367 nM (100 ng/mL) estradiol, live fluorescent images (D, F, H, J) and corresponding brightfield images (E, G, I, K) were taken at 3 d. 5xERE:GFPc262;esr1uab118 homozygous larvae (esr1 -/-) exhibit normal morphology, but lack fluorescence in heart valves, wheres heterozygotes (esr1 -/+) exhibit fluorescent heart valves. High magnification images of the heart are shown in H-K. Arrows indicate heart valves, arrow head indicates liver. Images are lateral views, anterior to the left, dorsal to the top. Scale bars, 500 μm (D-G), 100 μm (H-K). (PDF) [file pgen.1007069.s002.pdf]

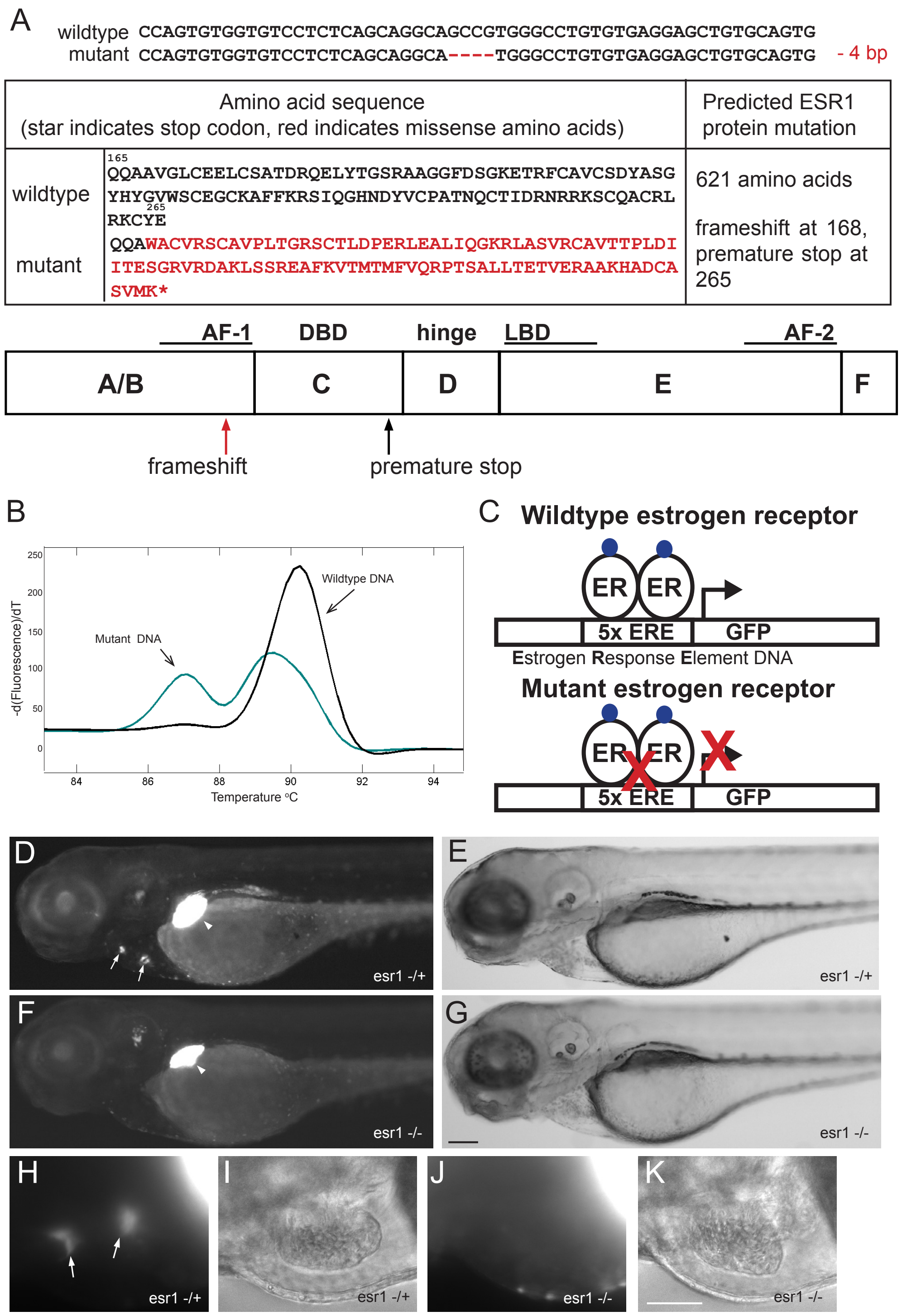
A
wildtype CCAGTGTGGTGTCCTCTCAGCAGGCAGCCGTGGGCCTGTGTGAGGAGCTGTGCAGTG
mutant CCAGTGTGGTGTCCTCTCAGCAGGCA----TGGGCCTGTGTGAGGAGCTGTGCAGTG
- 4 bp
Amino acid sequence
(star indicates stop codon, red indicates missense amino acids)
Predicted ESR1 protein mutation
wildtype
165
QQAAVGLCEELCSATDRQELYTGSRAAGGFDSGKETRFCAVCSDYASG
YHYGVWSCEGCKAFFKRSIQGHNDYVCPATNQCTIDRNRRKSCQACRL
265
RKCYE
621 amino acids
mutant
QQAWACVRSCAVPLTGRSCTLDPERLEALIQGKRLASVRCAVTTPLDI
ITESGRVRDAKLSSREAFKVTMTMFVQRPTSALLTETVERAAKHADCA
SVMK*
frameshift at 168, premature stop at 265
AF-1
DBD
hinge
LBD
AF-2
A/B
C
D
E
F
frameshift
premature stop
B
Wildtype DNA
Mutant DNA
-d(Fluorescence)/dT
Temperature °C
C
Wildtype estrogen receptor
ER
5x ERE
GFP
Estrogen Response Element DNA
Mutant estrogen receptor
D
E
F
G
H
I
J
K
esr1 -/+
esr1 -/-
