# Supplementary material for: G protein-coupled estrogen receptor regulates embryonic heart rate in zebrafish
Source: PLoS Genet. 2017 Oct 24;13(10):e1007069. doi: 10.1371/journal.pgen.1007069 (PMC5669493; doi:10.1371/journal.pgen.1007069)

A

wildtype CCTCTCCCCCATCTTCAACTCATCCTCTCCATCTCTGC
mutant CCTCTCCCCCA--TTCAACTCATCCTCTCCATCTCTGC -2bp

	Amino acid sequence (star indicates stop codon, red indicates missense amino acids)	Predicted ESR2A protein mutation
wildtype	22 VGGHILSPIFNSSSPSLPVENHPICIPSPYTDLGHDFSTLPFYSPALL GYSTSPLSDCSSVRQSLSPTLFWPPHSHVSSLTLQQQSRLQQ 112	554 amino acids
mutant	VGGHILSPIQLILSISASGESPHLHPIALHRPWPRLQHSALLQSRSAG VQHIAFIGLLVCAPVAKPDFILATSQPCFLTHIATTESTSTEPCY*	frameshift at 31, premature stop at 115

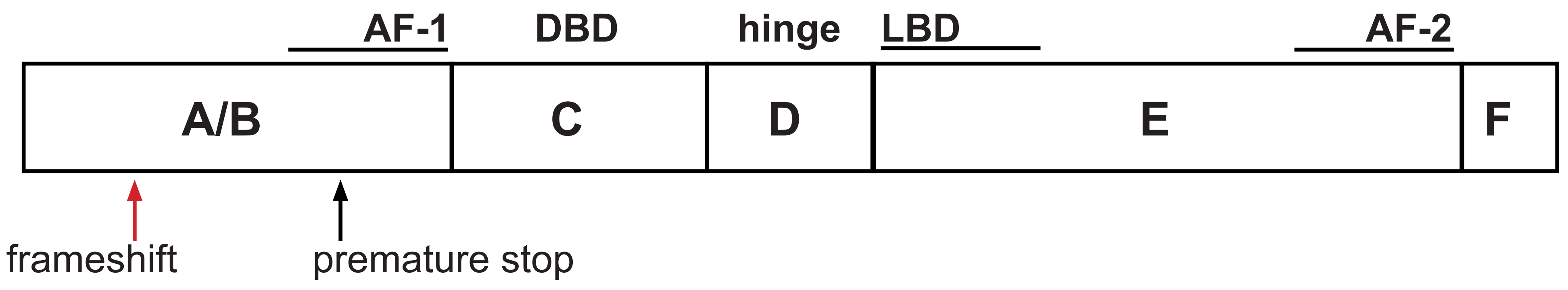

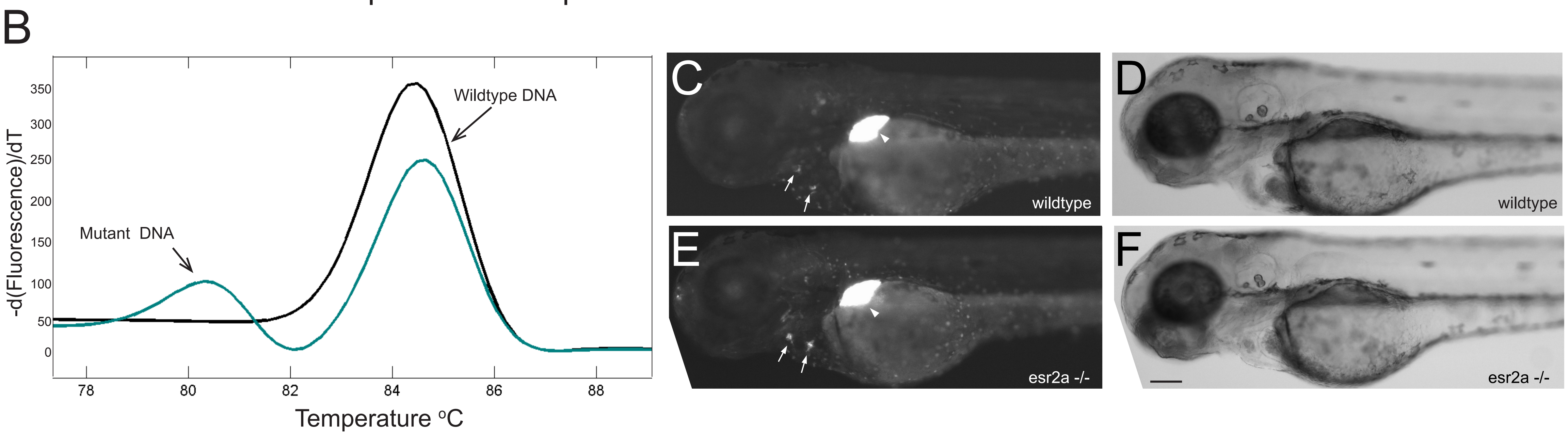

Supplement: S3 Fig — (A) Genomic DNA of esr2auab134 zebrafish contains an 2 basepair deletion (red dashes) in the esr2a coding region, resulting in a premature stop codon in the Esr2a (ERβ1) protein. Amino acid mutations are in red. Map indicates frameshift mutation and premature stop codon in the Esr2a protein. AF-1, activating function 1 domain; DBD, DNA binding domain; LBD, ligand binding domain; AF-2, activating function 2 domain. (B) High resolution melting curve analysis was used to distinguish mutants from wildtype. Curves represent DNA amplified from a wildtype AB (black) or esr2auab134 mutant zebrafish (cyan). (C-F) 5xERE:GFPc262 and 5xERE:GFPc262;esr2auab134 (esr2a -/-) 3-day post fertilization (d) larvae were exposed to 367 nM (100 ng/mL) estradiol. Live fluorescent images (C, E) and corresponding brightfield images (D, F) were captured at 4 d. esr2a -/- larvae exhibit normal morphology and fluorescence, consistent with data demonstrating that esr2a is not expressed during these developmental stages. Arrows indicate heart valves, arrow head indicates liver. Images are lateral views, anterior to the left, dorsal to the top. Scale bar, 500 μm. (PDF) [file pgen.1007069.s003.pdf]
