# Supplementary material for: G protein-coupled estrogen receptor regulates embryonic heart rate in zebrafish
Source: PLoS Genet. 2017 Oct 24;13(10):e1007069. doi: 10.1371/journal.pgen.1007069 (PMC5669493; doi:10.1371/journal.pgen.1007069)
Supplement: S4 Fig — (A) Genomic DNA of esr2buab127 zebrafish contains a 5 basepair deletion (red) in the esr2b coding region, resulting in a premature stop codon in the Esr2b (ERβ2) protein. Amino acid mutations are in red. Map indicates frameshift mutation and premature stop codon in the Esr2b protein. AF-1, activating function 1 domain; DBD, DNA binding domain; LBD, ligand binding domain; AF-2, activating function 2 domain. (B) High resolution melting curve analysis was used to distinguish mutants from wildtype. Curves represents DNA amplified from a wildtype AB (black) or esr2buab127 mutant zebrafish (cyan). (C-F) 5xERE:GFPc262;esr2buab127 3-day post fertilization (d) larvae were exposed to 367 nM (100 ng/mL) estradiol. Live fluorescent images (C, E) and corresponding brightfield images (D, F) were captured at 4 d. 5xERE:GFPc262;esr2buab127 homozygous larvae (esr2b -/-) exhibit normal morphology, but lack fluorescence in the liver. Arrows indicate heart valves, arrow head indicates liver. Images are lateral views, anterior to the left, dorsal to the top. Scale bar = 100 μm. (PDF) [file pgen.1007069.s004.pdf]

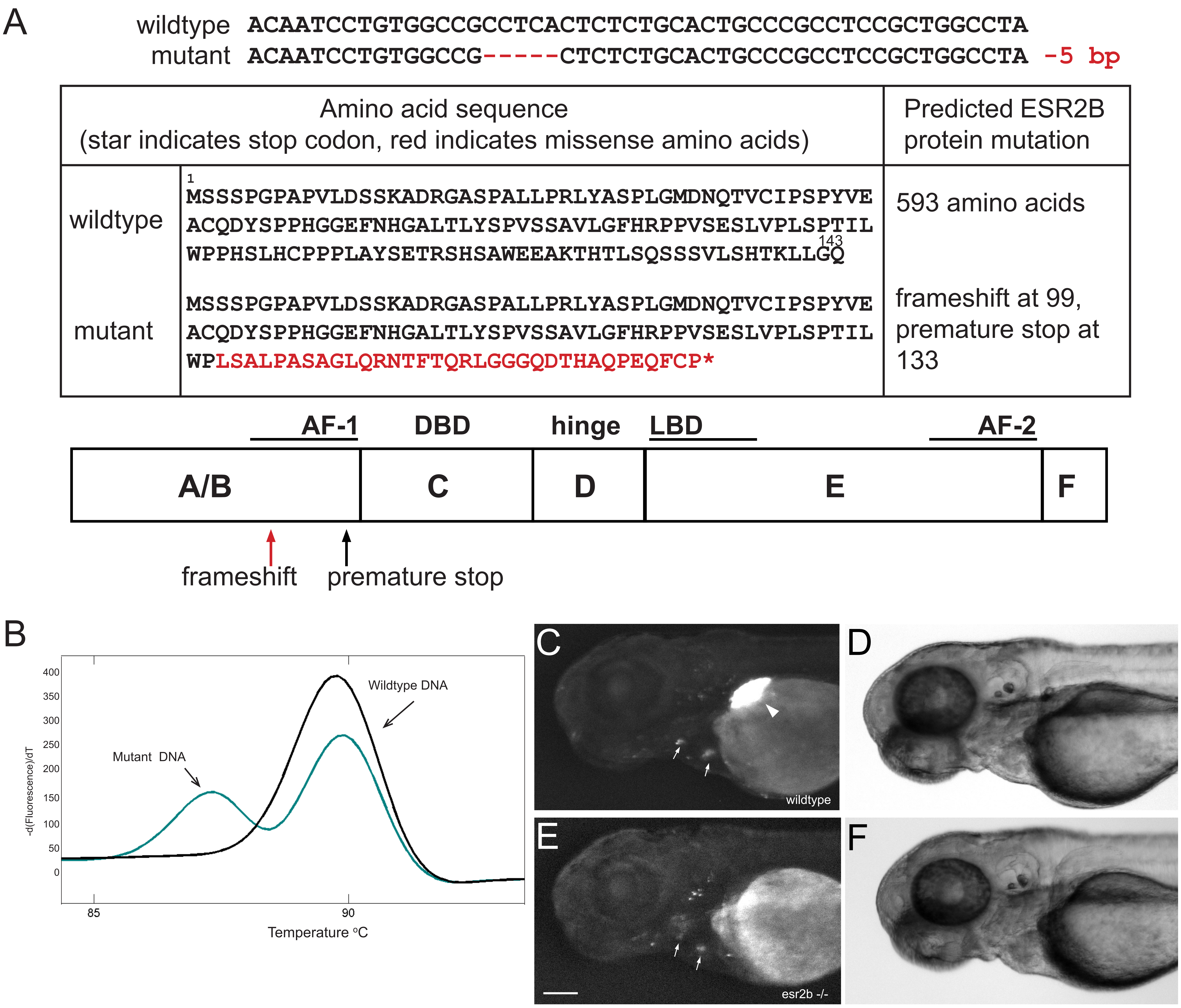
A
wildtype ACAATCCTGTGGCCGCCTCACTCTCTGCACTGCCCGCCTCCGCTGGCCTA
mutant ACAATCCTGTGGCCG-----CTCTCTGCACTGCCCGCCTCCGCTGGCCTA -5 bp
Amino acid sequence
(star indicates stop codon, red indicates missense amino acids)
Predicted ESR2B protein mutation
wildtype
1
MSSSPGPAPVLDSSKADRGASPALLPRLYASPLGMDNQTVCIPSPYVE
ACQDYSPPHGGEFNHGALTLYSPVSSAVLGFHRPPVSESLVPLSPTIL
WPPHSLHCPPPLAYSETRSHSAWEEAKTHTLSQSSSVLSHTKLLGQ
143
593 amino acids
mutant
MSSSPGPAPVLDSSKADRGASPALLPRLYASPLGMDNQTVCIPSPYVE
ACQDYSPPHGGEFNHGALTLYSPVSSAVLGFHRPPVSESLVPLSPTIL
WPLSALPASAGLQRNTFTQRLGGGQDTHAQPEQFCP*
frameshift at 99, premature stop at 133
AF-1
DBD
hinge
LBD
AF-2
A/B
C
D
E
F
frameshift
premature stop
B
-d(Fluorescence)/dT
400
350
300
250
200
150
100
50
0
Wildtype DNA
Mutant DNA
85
90
Temperature °C
C
wildtype
D
E
esr2b -/-
F
