# Supplementary figures and images for: G protein-coupled estrogen receptor regulates embryonic heart rate in zebrafish
Source: PLoS Genet. 2017 Oct 24;13(10):e1007069. doi: 10.1371/journal.pgen.1007069 (PMC5669493; doi:10.1371/journal.pgen.1007069)

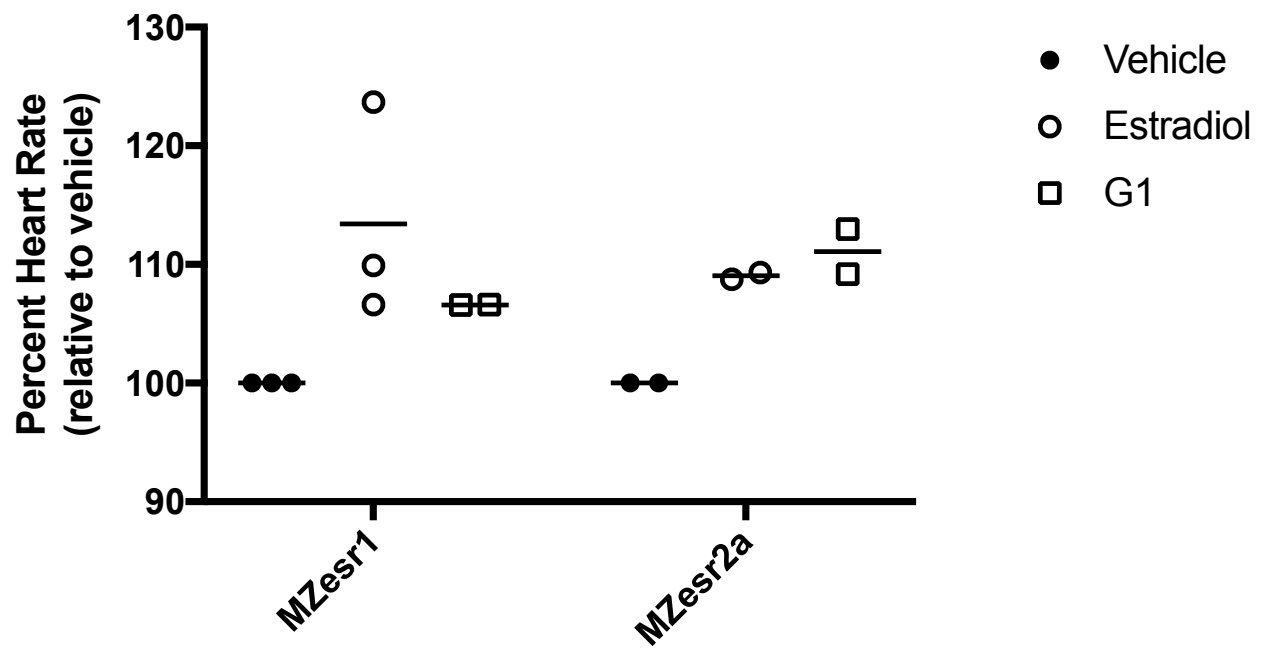

Supplement: S5 Fig — Maternal zygotic homozygous esr1 and esr2a mutant embryos (MZesr1, MZesr2a) were incubated in water containing estradiol (ER/GPER agonist, 3.67 μM), G1 (GPER agonist, 1 μM) or vehicle control (0.1% DMSO) at 49 hours post fertilization and heart rates were measured 1 hour post treatment. Each circle or square represents the mean heart rate from a single independent clutch of embryos (9–13 embryos per clutch). Horizontal lines are the mean of each treatment. (PDF) [file pgen.1007069.s005.pdf]

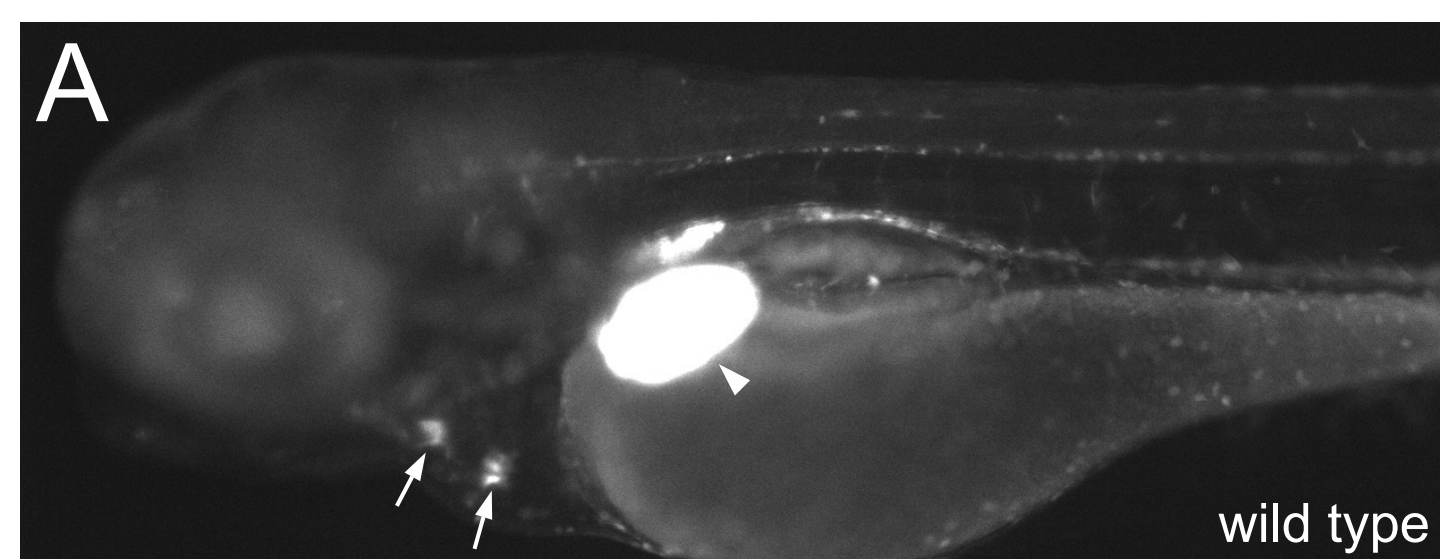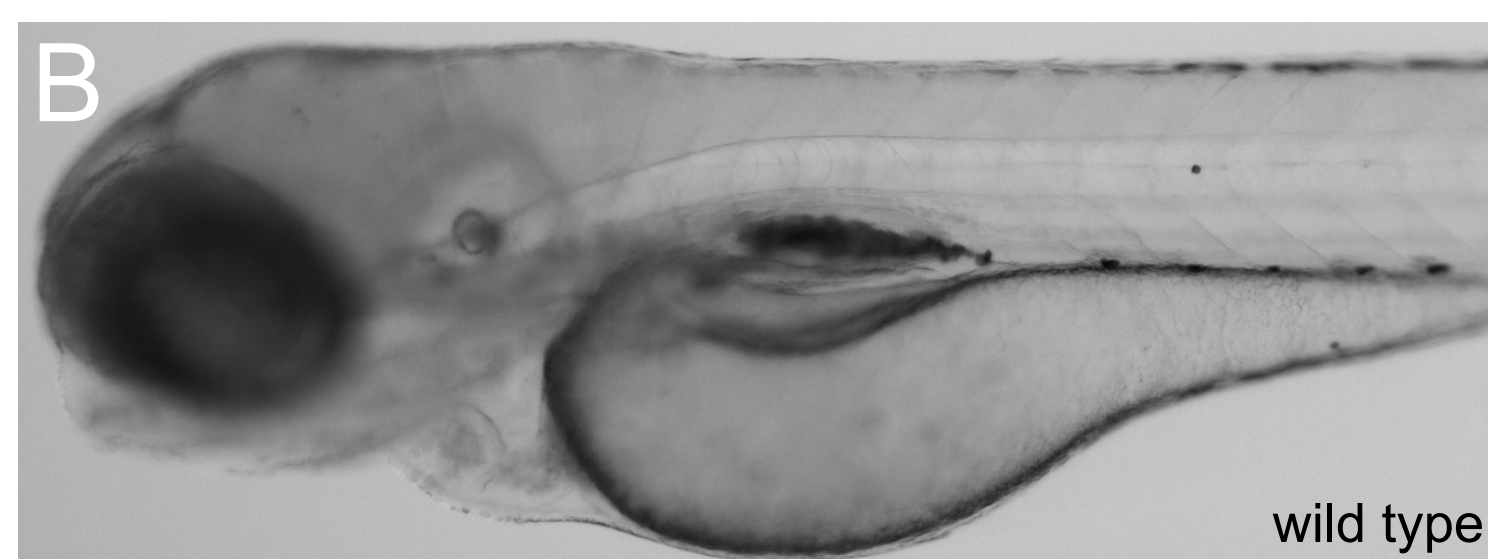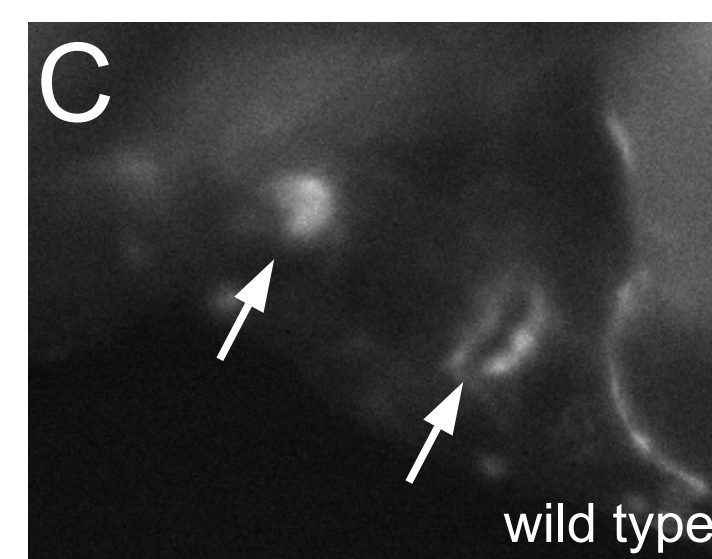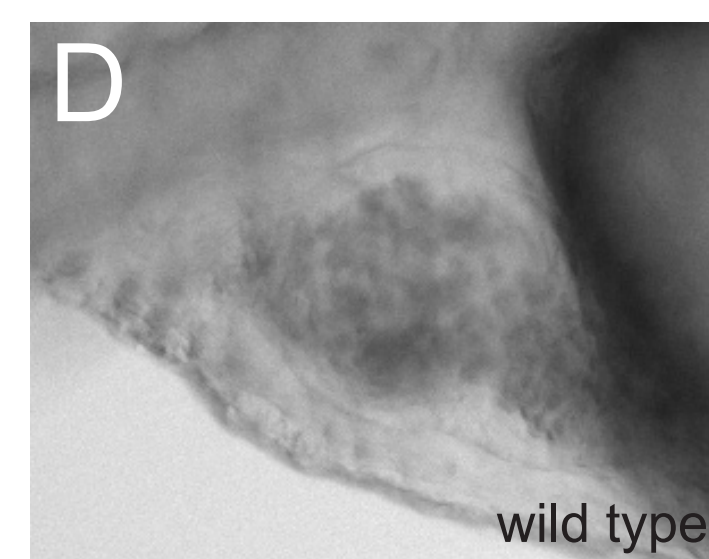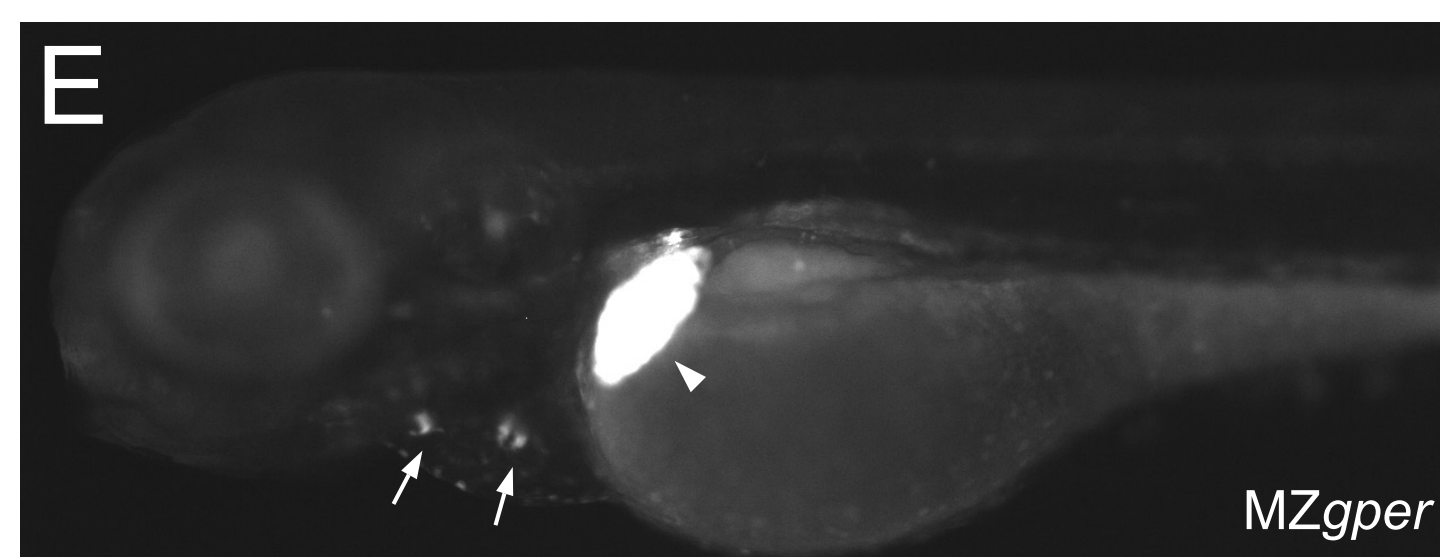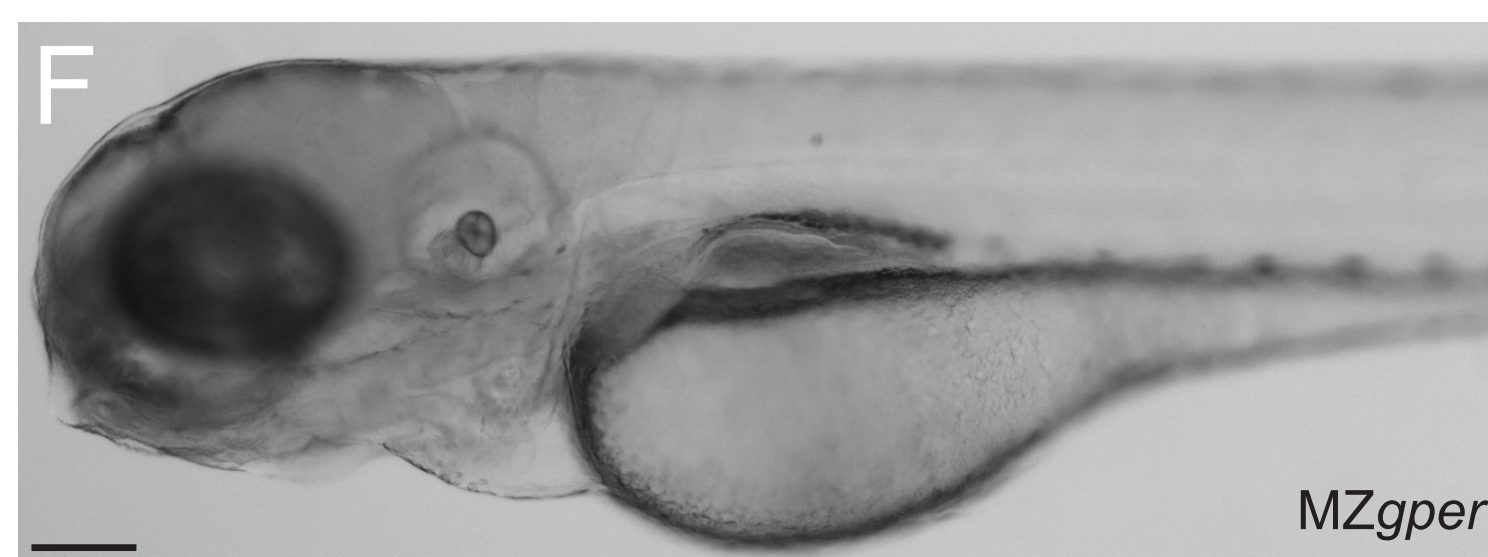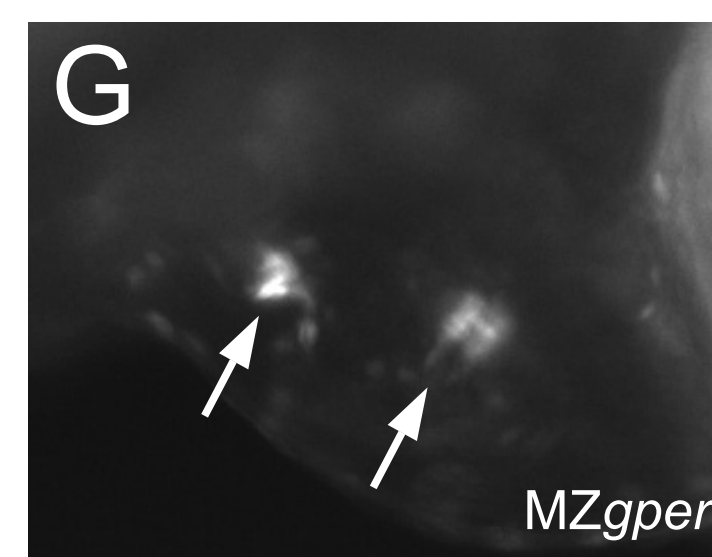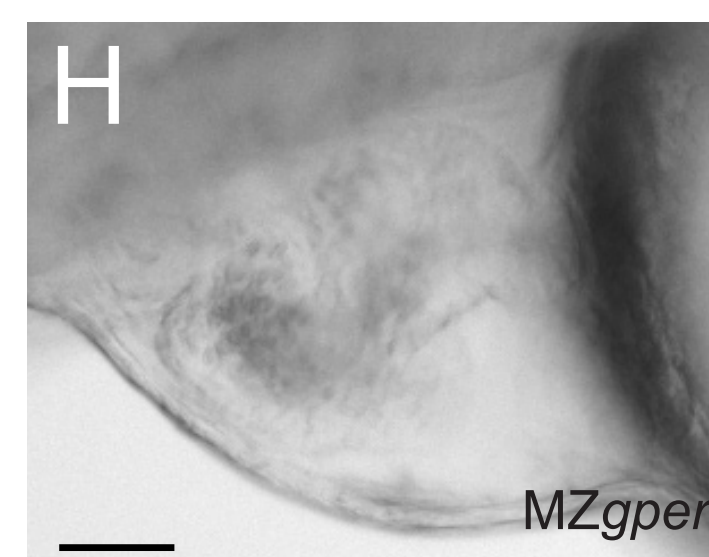

Supplement: S6 Fig — (A-H) Maternal zygotic gperuab102 homozygous larvae on the 5xERE:GFPc262 transgenic background (MZgper) were exposed to 367 nM (100 ng/mL) estradiol at 2-days post fertilization (2 d). Fluorescence (A, C, E, G) and corresponding brightfield images (B, D, F, H) were taken at 3 d. Fluorescence in the heart valves (arrows) and liver (arrow heads) is similar between MZgper and wild type. C, D, G, H, High magnification images of heart. Images are lateral views, anterior to the left, dorsal to the top. Scale bars, 500 μm (C-F), 100 μm (G-J). (PDF) [file pgen.1007069.s006.pdf]

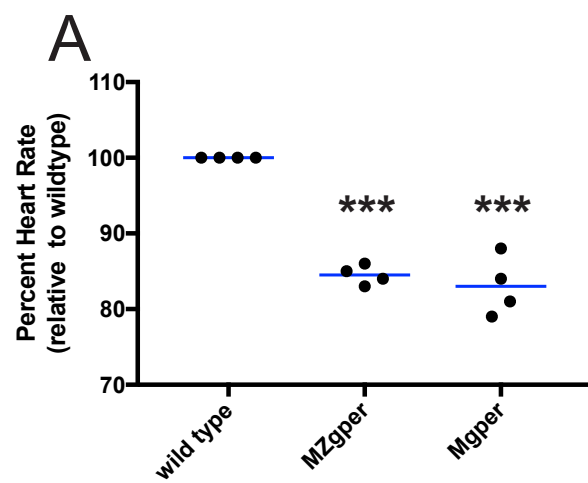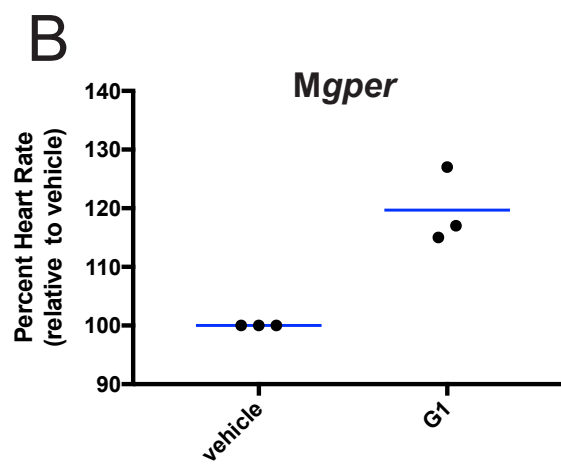

Supplement: S7 Fig — (A) Basal heart rate was measured at 51 hours post fertilization (hpf) in embryos reared in untreated water. Maternal zygotic homozygous gper mutants (MZgper), produced by breeding homozygous mutant females with homozygous males, and maternal gper mutants (Mgper), produced by breeding homozygous mutant females with wild-type males, had reduced heart rate compared to wild type. *** p<0.001 compared to wild type, two-way ANOVA. (B) Mgper embryos at 49 hpf were incubated in water containing G1 (GPER agonist, 1 μM) or vehicle (0.1% DMSO) and heart rate was measured 1 hour post treatment. G1 increased heart rate compared to vehicle, p<0.05, paired t test. Each circle represents the mean heart rate from a single clutch of embryos (6–12 embryos per clutch). Horizontal blue lines are the mean of each treatment or genotype. (PDF) [file pgen.1007069.s007.pdf]
